# The Causes and Prevention of Dental Caries

**Published:** 1885-01

**Authors:** Henry Sewill


ARTICLE IV

### THE CAUSES AND PREVENTION OF DENTAL CARIES.

Dr. Henry Sewill has written for the *Lancet* an article in which he sums up the causes and means of prevention of caries of the teeth. Caries is a process of disintegration, invariably commencing at the surface, proceeding inward, and due entirely to external agents; enamel and dentine are passive under this process. The predisposing cause of caries are: first, innate structural defects in the teeth, which render them more susceptible to the action of agents; secondly, all such diseases as are attended with vitiation of of the oral secretions, or which tend to the formation and deposit of acids and the accumulation of products of decomposition within the mouth; thirdly, to crowding and irregularity of the teeth, due to smallness and malformation of the maxillæ. The direct agents in initiating caries are acids, the products of chemical change and fermentation set up in fragment of organics matter which are commonly present in the mouth and lodge between the teeth. These acids are often assisted in their action by acid mucus secreted by unhealthy gums, acid saliva in some diseases, acid eructations from the stomach in others.

Enamel, according to Dr. Sewill, is always the starting point of caries. The enamel organ—that portion of a developing tooth from which enamel is formed—consists of epithelial cells, and it retains its epithelial character throughout the process of calcification. The completed tissue results from direct calcification of the internal layer of cells. Vascular papillæ arise from the contiguous layer of the dental sac, penetrate to a slight depth the external layer of cells, and convey nutriment to the developing tissue. On completion of the enamel these external cells, with the vascular loops, undergo atrophy and disappear.

Dentine consists of a homogeneous calcareous matrix with a basis of fibrous tissue. It is permeated by minute tubes occupied by fibrils which endow the tissue with sensibility, and which form the sole protoplasmic element in dentine. The bearing of these anatomical and physiological considerations upon the subject of caries depends upon the obvious fact that enamel is devoid of any physiological mechanism whereby either vital or pathological changes could be brought about in it, and that whatever changes enamel undergoes are induced by external agencies. It is a calcareous mass, devoid of cellular, elements, and incapable of imbibition. The one dark spot in our knowledge of the remote causation of caries, according to Sewill, is found in the first predisposing cause: inherent structural weakness of the tissues. The enamel varies indefinitely, in different individuals, in the quality of its calcareous matrix; we often, in fact, encounter teeth whose soundest enamel is chalklike in its softness. Teeth of general defective character often present patches of surface, the enamel of which consists merely of an imperfectly united mass of granules. Sometimes, in places, enamel fibres are penetrated at their centres by minute tubes which render the tissue at such places porous. Honeycombed teeth have enamel full of small pits and fissures, and there is scarcely a set of teeth to be found in which here and there, in one or more teeth, an isolated crack or cleft, a solution of continuity of the tissue, does not exist.

While our knowledge of origination of mal-development of teeth is very incomplete, there is evidence that in all races of advanced civilization there is a notable deterioration in dental development. Comparative disease of the organs of mastication has led in civilized races to decrease in size and strength of the whole apparatus of mastication—jaws, muscles, and teeth. This fact has been demonstrated by Professor Flower, who shows by examination and measurement of many thousand skulls that there is a gradual diminution in the size of the teeth from the anthropoid apes through the lower races of man to the European. It has been argued that progressive dental deterioriation may be brought about by the overwhelming demand upon upon the vital powers by the growth of the brain and its increased exercise in modern life. The brain and the jaws are alike fed from the common carotid artery, and it is urged that the demand for blood by the growing and working brain leads to imperfect supply of the masticatory organs. Again, dental deterioration has been ascribed to improper and imperfect feeding of children, to insufficiency in the the food of lime salts, the proper pabulum of developing teeth. If this were true the whole osseus system might be expected to be ill-developed, and it would be impossible to find a single individual with a solid skeleton. Perhaps, with very rare exceptions, there is no modern people displaying general progressive physical degeneration, and which is accompanied by ill-developed teeth. But it must be recollected that there has been among the population a constant augmentation in the number of the physically imperfect. Vast numbers of the phthisical, the scrofulous, the rickety, and congentially syphilitic who would formerly have perished in childhood, are now by science kept alive, and these imperfect types are all associated with faulty dental development.

With regard to the association of defective dental tissues with particular diatheses, we find ourselves on firmer ground. Hereditary syphilis leads often to the imperfect formation of tooth tissues. The children of syphilitic parents are apt to have the enamel soft and easily broken down.

Imperfect dental development is also found in the majority of cases of scrofula. One class of patients, the subject of phthisis of tubercular origin, almost invariably presents faulty dental tissues. This is the type most frequently found in females, in which there is often, with a fragile form, considerable facial beauty, iu which the eyes are large and expressive, the complexion fair, with the blue veins visible beneath the skin. In this not uncommon type Dr. Sewill has invariably found the teeth, though well shaped and fair to look at, covered with the softest enamel. With rickets, too, inherently defective teeth are invariably found.

Premising that but little can be done to prevent dental deterioration, except by the promotion of national sanitation in the broadest sense, and that any treatment designed to inflnence beneficially the developing teeth of the fœtus in utero when the parents nave been the subjects of the diatheses or cachexiæ above mentioned can be of little effect, the writer considers other factors in the causation of caries, *viz.*, vitiation of the buccal secretions. The diseases of our times are largely those which give rise to derangement of these secretions, for many or most of them are either diseases of the digestive organs or maladies accompanied by disturbance of the organs of digestion. In this way we may account for the prevalence of caries among certain portions of the community whose dental tissues are not of tho worst structure The constant presence of dyspeptic troubles in some classes of factory operatives is undoubtedly enough to account for the rapid tooth decay from which they suffer. Moreover, some of the diatheses, notably a variety of scrofula attended with a chronic condition of congested oral mucous membrane, and rickets, are often accompanied by a secretion of viscid saliva and acid dyspepsia. The broad facts now before us are: first, that without acid, without vitiation of the secretions of the mouth, caries is impossible; and secondly, that acid capable of dissolving enamel—and of more rapidly dissolving it in proportion as the tissue is soft and ill-made—is always being formed in greater or less quantity in every mouth in which absolute cleanlinees does not exist.

In combating this second cause of caries, the patient's general health must first be considered, and we must remember that probably every lapse from a perfect standard will be attended with a proportionate vitiation of the secretions of the mouth. The question of the general health should then first receive attention and dyspeptic troubles be remedied. But even with perfect health and perfect dental tissues caries may appear if organic debris be allowed to remain and decompose in contact with the teeth. The first thing, therefore in every case is to insure mechanical cleanliness by the use of a tooth-brush and tooth-pick, which latter should be of quill or wood only. When the teeth are crowded or of delicate structure, the use of floss silk passed between the teeth and rubbed to and fro supersedes the use of the tooth-pick. The tooth powder recommended by Dr. Sewill is as follows: Take of Castile soap, two drachms, powdered orris-root, half an ounce, borax, two drahms, and prepared chalk, two ounces. Mix and rub together; flavor with a few drops of oil of cloves and lavender. Instead of the perfume, Dr. Sewill has lately added a few drops of carbolic acid to each ounce of the powder, the whole being flavored with eucalyptus oil. The mixture should be used freely with a wet tooth-brush, the mouth to be freely rinsed with water at intervals during the brushing. In all cases where great vitiation of the secretions is present, as in the dyscrasia of pregnancy, extra means should be adopted to prevent putrefaction and fermentation in the deposits which form upon the teeth. A useful mixture for this purpose is made by dissolving a grain of bichloride of mercury in an ounce of eau de Cologne or tincture of lemon. Mix a teaspoonful of this with two-thirds of a wineglassf of water and rinse the mouth with it several times a day.

As for the caries which depends on crowded jaws and irregularity of the teeth (favoring the retention of food-debris in contact with the teeth), the most beneficial measure to prevent caries in these cases, especially when the teeth are of imperfect formation, is the extraction of the six-year-old molars as soon as the second molars are well in place. The first permanent molars are generally the worst-made teeth of the whole set, and in cases where room is urgently needed it is seldom that their preservation is possible. Removal of these teeth allows an equal and sufficient spreading apart of all the teeth and prevents crowding. The operation of filing the contiguous surfaces of canines and incisors as an effectual means of cutting short the progress of incipient caries in crowded jaws is too well recognized to need comment.—*Medical Record.*

